# A new species of *Metagovea* Rosas Costa, 1950 from Napo Province, Ecuador (Opiliones, Cyphophthalmi, Neogoveidae)

**DOI:** 10.3897/zookeys.477.8706

**Published:** 2015-01-26

**Authors:** Alessandro P. L. Giupponi, Adriano Brilhante Kury

**Affiliations:** 1 Laboratório de Referência Nacional em Vetores das Riquetsioses, LIRN-FIOCRUZ, Manguinhos, Rio de Janeiro-RJ, CEP 21040-360, Brazil; 2 Museu Nacional, Universidade Federal do Rio de Janeiro, Laboratório de Aracnologia, Quinta da Boa Vista s/n, São Cristóvão, Rio de Janeiro-RJ, CEP 20940-040, Brazil

**Keywords:** Neotropical fauna, conservation, Ecuador, taxonomy, harvestmen

## Abstract

As a result of an expedition to Ecuador in 2014, a new species of mite harvestman was discovered. This new species belonging to the genus *Metagovea* Rosas Costa, 1950 – *Metagovea ligiae* **sp. n.** – is described, based on male and female specimens from Napo Province, Ecuador. This is the fourth species described for the genus and the second from Ecuador. A simple terminology is proposed for the microtrichiae of the spermatopositor and genital characters in the family are discussed. The genus *Brasiliogovea* Martens, 1969 is consistently misspelled in the literature as *Brasilogovea*. The description of *Metagovea ligiae* offered opportunity to discuss some aspects of systematics of the family.

## Introduction

Cyphophthalmi is the least diverse suborder of Opiliones arachnids, and is represented in tropical and temperate ecosystems worldwide. It includes six families and around 200 described species, although this number is heavily underestimated (Kury 2013; Giribet 2000; 2000+). The smaller diversity of Cyphophthalmi rests in the New World, with little more than 30 described species.

Neogoveidae has 26 species arranged in 8 genera, mostly Neotropical, but also Nearctic and Afrotropical (Rosas Costa 1950; Giribet 2000; DaSilva et al. 2010; Benavides and Giribet 2013; Karaman 2013; Clouse and Wheeler 2014): *Brasiliogovea* Martens, 1969 (2 spp.), *Canga* DaSilva, Pinto-da-Rocha & Giribet, 2010 (1 sp.), *Huitaca* Shear, 1979 (7 spp.), *Metagovea* Rosas Costa, 1950 (3 spp.), *Neogovea* Hinton, 1938 (5 spp.), *Tucanogovea* Karaman, 2013 (1 sp.), *Metasiro* Juberthie, 1960 (3 spp.), *Parogovia* Hansen, 1921 (3 spp.) and also Genus? *enigmaticus* Martens, 1969.

The genus *Brasiliogovea* Martens, 1969 is consistently misspelled in the literature as “*Brasilogovea*”, beginning with Shear (1980), including the important Giribet’s (2000) catalogue through the recent publications (Benavides and Giribet 2013; DaSilva et al. 2010; Karaman 2013). Both in Neave’s Nomenclator (Neave 2005) and in Zoological Record (ZSL 1871+), *Brasiliogovea* is correctly spelled, but in Hallan’s synopsis (Hallan 2005), *Brasiliogovea* is misinterpreted as a misspelling of the Zoological Record.

*Metagovea* is only known from South America, in the Andean and Amazonian regions. There are only three described species, but a plethora of undescribed species are already known (Benavides and Giribet 2007): *Metagovea disparunguis* Rosas Costa, 1950 (Colombia), *Metagovea oviformis* Martens, 1969 (Brazil: Manaus) and *Metagovea philipi* Goodnight & Goodnight, 1980 (Ecuador). In the present work a fourth species of *Metagovea* is described from Pacto Sumaco, in the Napo Province, in the eastern Andean slope.

## Methods

The specimens were collected during 15th–16th February 2014 through meticulous visual search throughout the floors of the forest and buildings. All specimens were captured with a fine brush and placed in vials containing 75% and 100% ethanol.

Nomenclature of body parts and measurements follows the model of Benavides and Giribet (2013). Terminology of the structures of spermatopositor follows Juberthie (1970; 1979) and Karaman (2013), with some modifications: (1) recognition of apical movable fingers (**dma**, *digitus mobilis apicalis*) which might not be homologous with **dml** (*doigt mobile latéral*) of Juberthie/Karaman, and (2) naming of three groups A, B, C of microtrichiae, hitherto unnamed, which are clearly recognizable also in other families of Cyphophthalmi (Fig. 6).

The following abbreviations are used: MNRJ = Museu Nacional, Universidade Federal do Rio de Janeiro, Brazil; QCAZ = Museo de Zoologia, Pontifícia Universidad Catolica del Ecuador – Quito, Ecuador; MCZ = Museum of Comparative Zoology, Harvard University, Cambridge, Massachusetts, USA.

Illustrations of the spermatopositor and ovipositor were made through a Carl Zeiss Primo Star microscope and AxioVision LE image capture system, with the stacking software Combine ZP Suite (by Alan Hadley). SEM images were made at JEOL JSM-6390 LV.

## Taxonomy

### Family Neogoveidae Shear, 1980

#### Genus Metagovea

Taxon classificationAnimaliaOpilionesNeogoveidae

Rosas Costa, 1950

##### Type species.

*Metagovea disparunguis* Rosas Costa, 1950, by original designation.

##### Included species.

*Metagovea disparunguis* Rosas Costa, 1950 (Colombia), *Metagovea ligiae* sp. n. (Ecuador), *Metagovea oviformis* Martens, 1969 (Brazil: Manaus) and *Metagovea philipi* Goodnight & Goodnight, 1980 (Ecuador).

#### Metagovea ligiae sp. n.

Taxon classificationAnimaliaOpilionesNeogoveidae

http://zoobank.org/469C4A18-15B6-45AC-8F94-81B2D675F492

Figs 1 , 2 , 3 , 4 , 5 , 6 , 7 , 8

##### Etymology.

The new species is named after friend and fellow arachnologist Ligia Benavides for her work on Neotropical Neogoveidae.

##### Type material.

♂ holotype: Ecuador, Napo, Sumaco-Galeras National Park, Pacto Sumaco (-0.66577°, -77.59813°, 1526 m), 15–16 February 2014, A.B. Kury and A.P.L. Giupponi leg.; ♀ (1) paratype: same data as holotype (QCAZ 322). ♂ (1) and ♀ (1) paratypes: same data as holotype (MCZ 45452); ♂ (3, of which 1 mounted for SEM) and ♀ (8, of which 1 mounted for SEM) paratypes: same data as holotype (MNRJ 8434).

##### Diagnosis.

**Figure 1.**
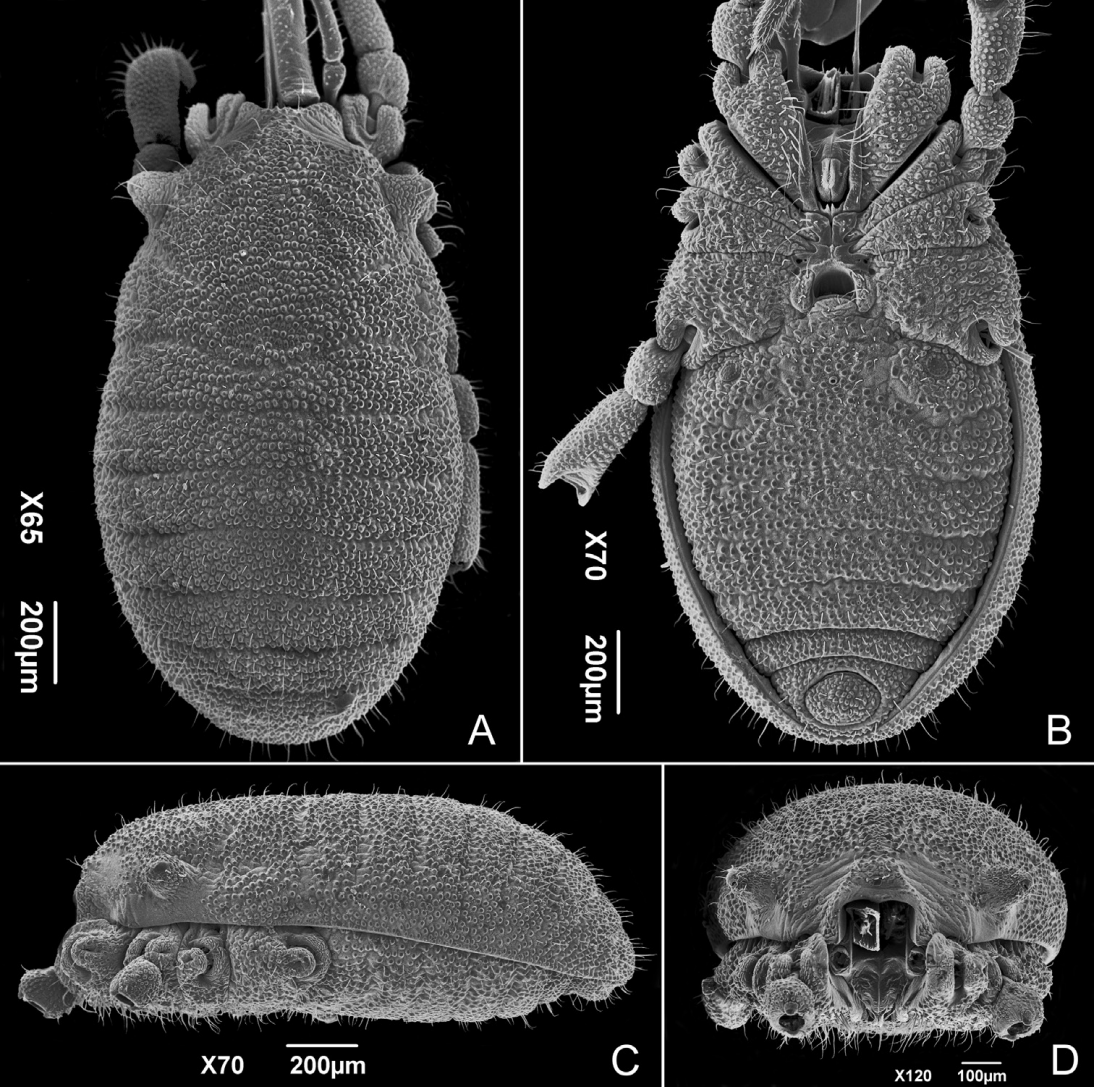
*Metagovea ligiae* sp. n., male paratype (MNRJ 8434). **A** Habitus, dorsal view **B** Same, ventral view **C** Same, without appendages, lateral view **D** Same, frontal view.

**Figure 2.**
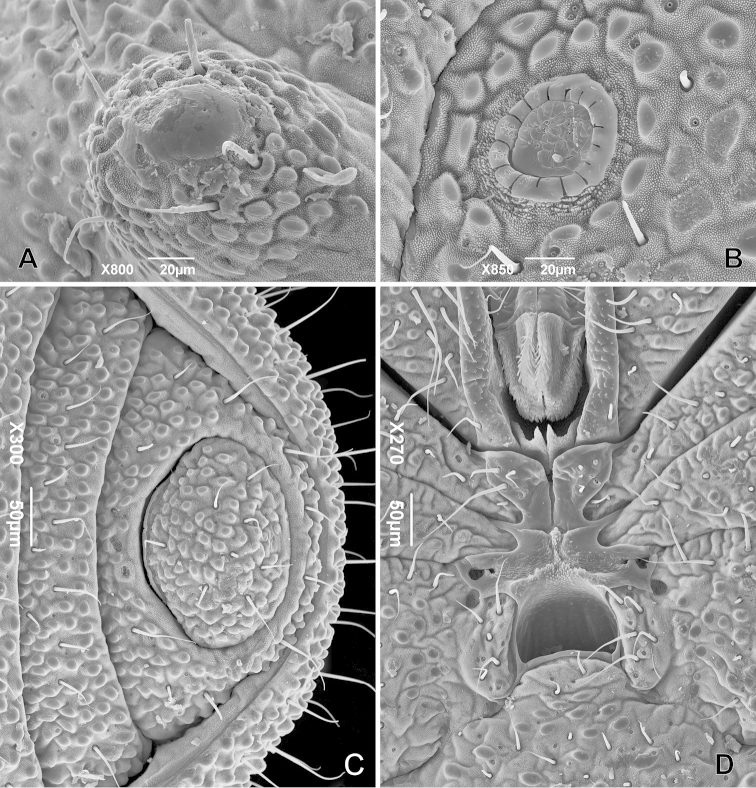
*Metagovea ligiae* sp. n., male paratype (MNRJ 8434). **A** Ozophore, oblique view **B** Tracheal stigma, ventral view **C** Anal region **D** Ventral prosomal complex.

Small animals, maximum body length 1.5 mm; body outline on dorsal view oblong (Fig. 1A); eyes and eye lenses absent (Figs 1A, C, D); spiracles circular (Fig. 2B) (*sensu* Giribet and Boyer 2002: 115); ventral prosomal complex with coxae II-IV fused, coxae I free, sternum absent, area of contact with coxal lobe III forming a complex arch delimiting the coxal pores (Figs 1B–2D); gonostome semicircular with concave posterior margin (Fig. 2D); ventral opisthosomal region with anal glands on sternal part of corona analis (Figs 1C–2C); spermatopositor with two pairs of shorter robust microtrichiae A, four pairs of much elongate microtrichiae B, three pairs of subapical microtrichiae C not as long as B, two pairs of movable fingers: small apical **dma** located between left and right groups of microtrichiae C; much larger **dmm**, arising from dorso-medial lobe. *Metagovea ligiae* may be distinguished from *Metagovea disparunguis* and *Metagovea oviformis* by the body longer than 1.4 mm and adenostyle sinuous changing curvature (Fig. 4D) instead of parabolic. *Metagovea ligiae* may be distinguished *Metagovea philipi* by the much shorter basichelicerite, with only one ventral protuberance; pedipalpal trochanter clearly shorter than femur and incrassate distally; femur III dorso-apically with a protuberance; adenostyle double curved, single-pointed, pointing distally.

##### Description of male.

**Measurements.** Male holotype: total length: 1.5 mm, greatest width: 0.8 mm, in the posterior part of prosoma; length/width ratio: 1.88; length of chelicerae: 1.0 mm, pedipalps (trochanter to tarsus): 1.0 mm; legs I: 1.5 mm, II: 1.2 mm, III: 1.0 mm, IV: 1.3 mm.

**Color (in ethanol) and tegument.** Body and appendages dark brown with most of dorsal and ventral surfaces and legs showing a dense tuberculate-microgranulate structure (Murphree 1988: 239).

**Body** (Fig. 1A). Prosomal region occupying less than half of the body size (Fig. 1A). Anterior margin of dorsal scutum with a pair of processes lateral to chelicerae. Lateral margin of prosoma bulging considerably behind ozophores, at widest part of body. Eyes and eye lenses absent. Ozophore conical of type 2 (*sensu* Juberthie 1970: 1373; i.e. dorso-laterally oriented) (Figs 1A, C, D, 2A). Dorsal scutum without special modifications. Opisthosomal mid-dorsal longitudinal sulcus absent (Fig. 1A).

Ventral prosomal complex (Fig. 1B) with coxae II-IV fused, coxae I free, sternum absent, coxae IV separated by gonostome (Fig. 1B); gonostome semicircular with concave posterior margin (Fig. 2D). Coxal lobes I much longer than wide, narrower anteriorly, subparallel, each armed with 2 posterior setae. Coxal lobes II anteriorly extremely thin, abruptly widening until mid-length where they start to narrow posteriorly, with 4 setae on wider part. Coxal lobes III longer than main part of coxal lobes IV; coxal lobes IV coarsely spiked in the middle, forming anterior-lateral margins of the gonostome. On the area of contact with coxal lobe III forming a complex arch delimiting the coxal pores. Coxae II-IV with rounded glandular fields at the concave part of respective coxal lobes.

Spiracles circular (Fig. 2B) (*sensu* Giribet and Boyer 2002: 115). Ventral opisthosomal region with anal glands on sternal part of corona analis (Fig. 2C, see also female). Opisthosomal tergite IX and sternites 8 and 9 fused into a corona analis (Fig. 2C). Anal plate oval. This and sternites densely covered by small conical granules and large flattened tubercles, some of them pectinate (Fig. 2C, see also female).

**Figure 3.**
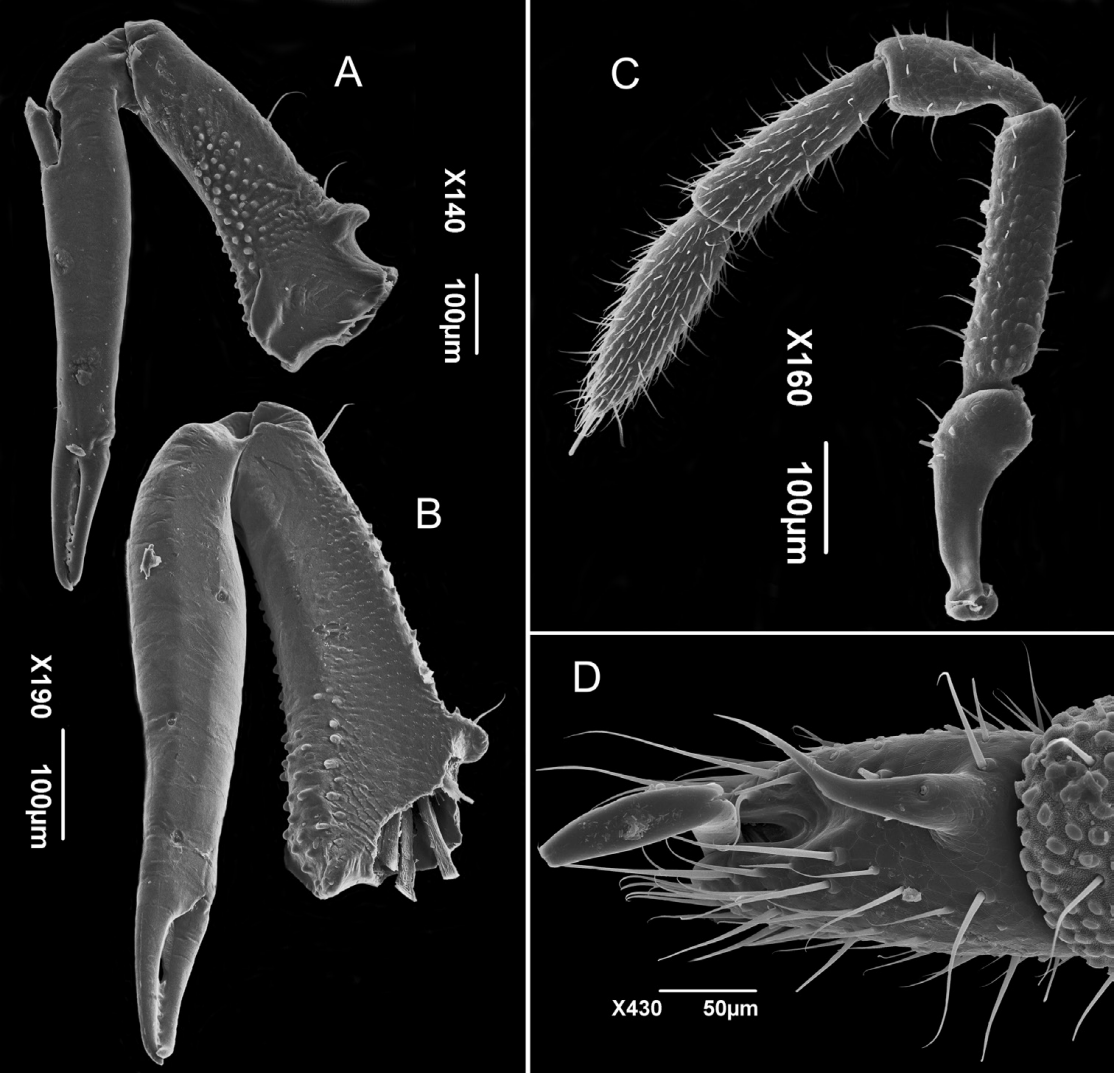
*Metagovea ligiae* sp. n., male paratype (MNRJ 8434). **A** Left chelicera, ectal view **B** Right chelicera, mesal view **C** Left pedipalpus, ectal view **D** Left tarsus IV, dorsal view.

**Chelicera** (Fig. 3A–B) elongate, with few and spaced dorsal setae; non-protruding type (sensu Giribet 2003). Basichelicerite with ectal surface granular, denser than ventro-mesal surface, but mesal with scale-bristles; dorso-mesal granules; with conspicuous dorsal crest and without ventral process. Second article elongate, widest near the base, without ornamentation. Dentition concentrated at the ends of the both cheliceral fingers.

**Pedipalp** (Fig. 3C) Trochanter unarmed, with few ventro-distal setae, much thickened at distal third, doubling its height. Femur cylindrical, with few rows of setae, all over its length; surface coarsely granulose, more so on basal and middle thirds. Patella thin on basal third, abruptly thickening in middle third up to the apex where it is twice as thick as basal third. Tegument smoother than femur and setation pattern similar to it. Tibia with abundant rows of setae, much denser than basal articles, slightly thinner at base, gradually thickening to apex. Tarsus fusiform, still more densely setose than tibia, ending in straight tubular claw.

**Figure 4.**
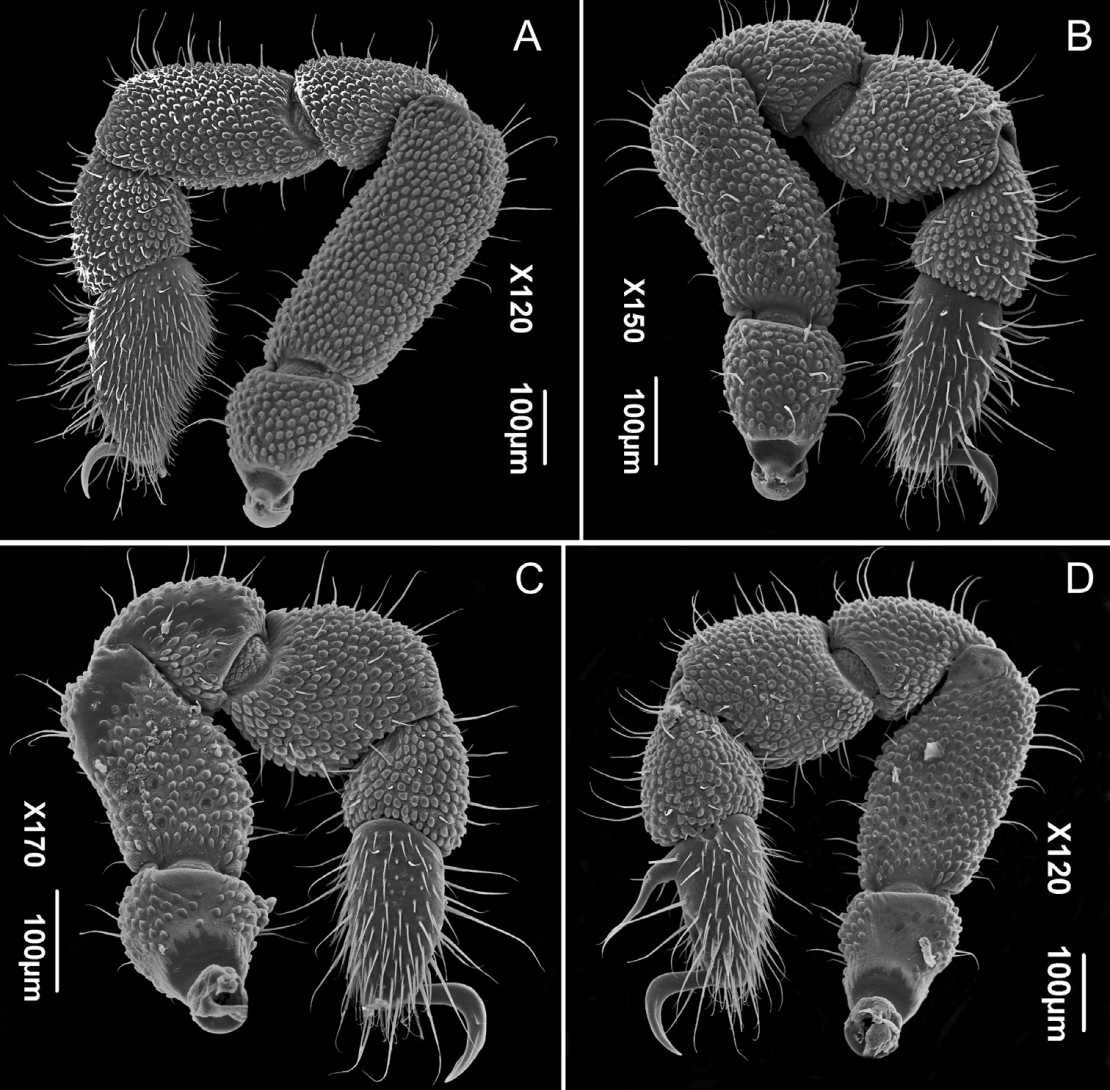
*Metagovea ligiae* sp. n., male paratype (MNRJ 8434). **A** Left leg I, retrolateral view **B** Right leg II, retrolateral view **C** Right leg III, retrolateral view **D** Left leg IV, retrolateral view.

**Legs** (Figs 4A–D). Robust, leg formula I, IV, II, III. Trochanter to metatarsus of legs I-IV densely granulated, less on Tr-Pa III, Tr IV, smooth on Ta I–IV. All articles setose, density of setae increasing distally, reaching maximum on tarsi I–IV. Tarsus I with a distinct solea (subapical modification where sensory hairs concentrate, Fig. 4A) taking up about 2/3rds of the tarsus length. Tarsus of leg IV undivided (Fig. 4D), with a lamelliform elongate, sinuous and acuminate adenostyle, positioned basally on the dorsal side on tarsus IV (Figs 3D–4D). Claw of leg II (Figs 4B). With a distinct row of five teeth. Claws of legs III-IV beveled laterally.

**Figure 5.**
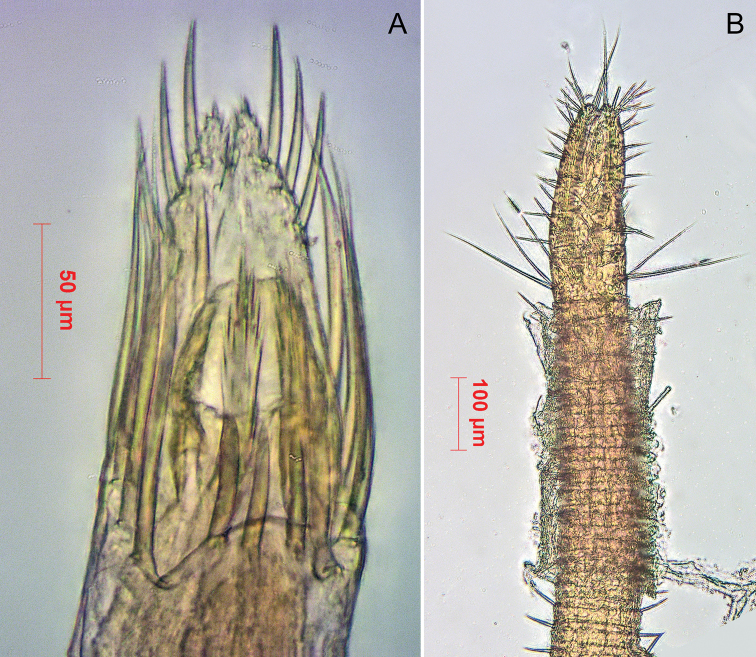
*Metagovea ligiae* sp. n., male paratype (MCZ 45452). **A** Spermatopositor, dorsal view **B** Female paratype (MCZ 45452), ovipositor, lateral view.

**Figure 6.**
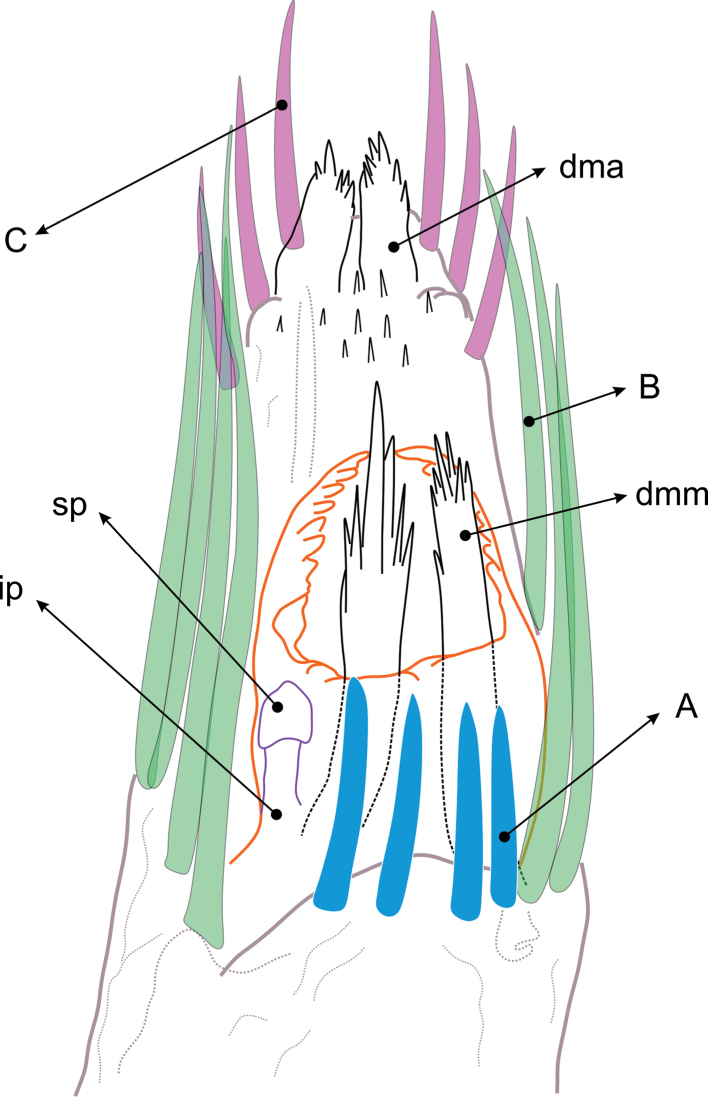
*Metagovea ligiae* sp. n., male paratype (MCZ 45452). Spermatopositor, interpretative drawing of photograph in Figure 5A. Abbreviations: **A** = microtrichiae A **B** = microtrichiae B **C** = microtrichiae C **dma** = digiti mobiles apicales **dml** = digiti mobiles laterales **ip** = inner papilae **sp** = sensory papilae.

**Spermatopositor** (Figs 5A, 6). Two pairs of shorter robust microtrichiae A close together on a proximal lobe. Four pairs of microtrichiae B much elongate, on the laterals. Three pairs of subapical microtrichiae C not as long as B. Two pairs of terminally fimbriate movable fingers: small apical **dma** located between left and right groups of microtrichiae C; much larger **dmm**, arising from dorso-medial lobe. More basally, near the genital orifice, a pair of sensory papillae and another of inner papillae.

##### Distribution.

Known only from the type locality, Pacto Sumaco, Napo, Ecuador (Fig. 9).

##### Female

**Figure 7.**
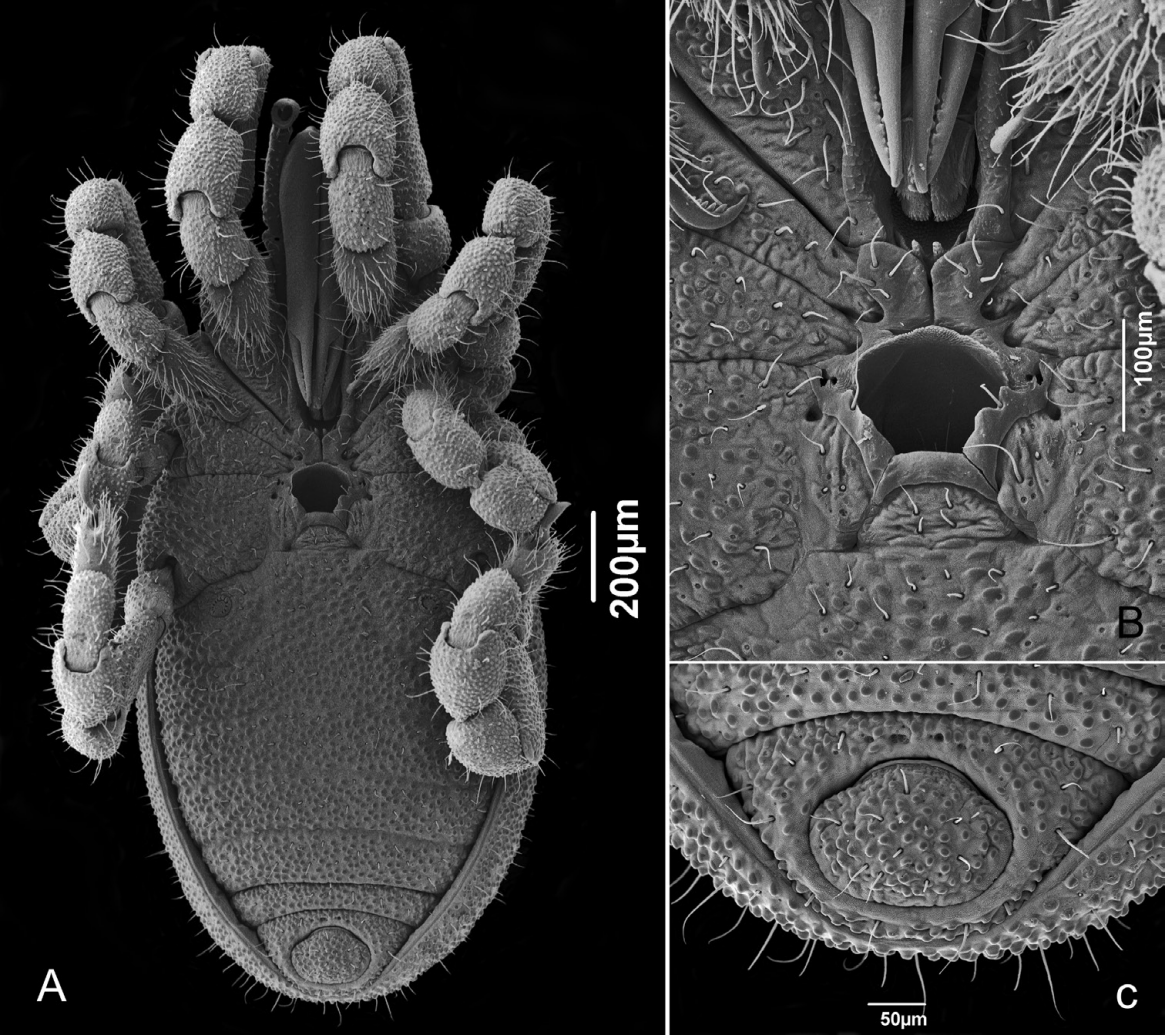
*Metagovea ligiae* sp. n., female paratype (MNRJ 8434). **A** Habitus, ventral view **B** Ventral prosomatic complex **C** Anal region.

**Figure 8.**
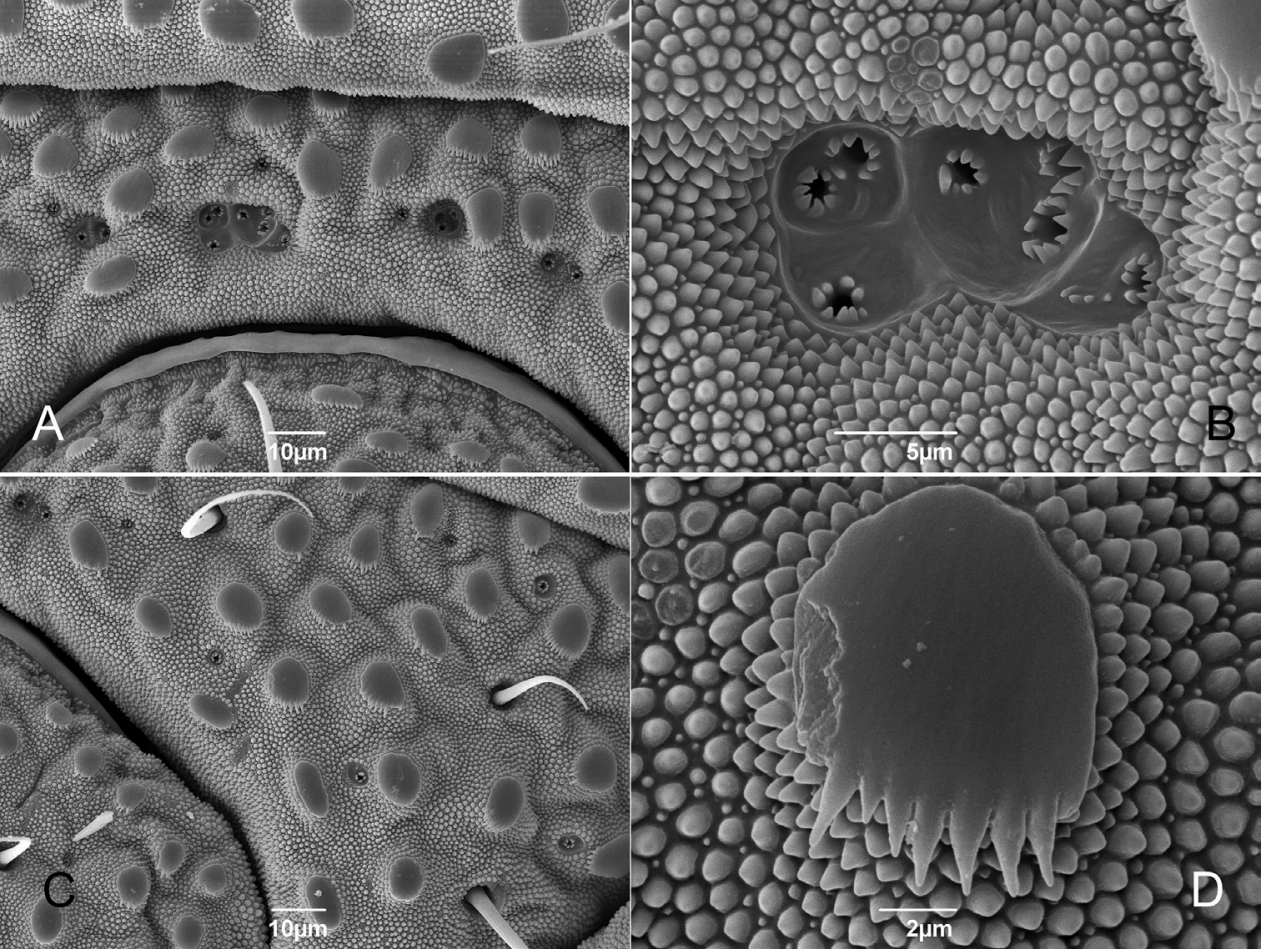
*Metagovea ligiae* sp. n., female paratype (MNRJ 8434). **A** Corona analis, antero-median region with clustered anal glands **B** Same, detail of the anal glands **C** Corona analis, left antero-lateral region with isolated anal glans **D** Same, detail of the pectinate tubercle.

(Figs 7–8). Ventral opisthosomal region with anal glands on sternal part of corona analis as in male, consisting of isolated (on laterals, Fig. 8C) and clustered pores (Figs 8A–B). Sternal large tubercles often pectinate (Fig. 8D). Gonostome pentagonal, with coxal lobes IV and posterior margin of gonostome much wider than in male (Fig. 7B).

##### Natural history.

All specimens were collected in an area of ​​about 10 m^2^, under a house built partly on a small slope in a nature conservation area (Fig. 10A), but with a strongly disturbed secondary forest. The specimens were found beneath stones, wood and other “rubbish” left on the ground of sometimes compacted, sometimes loose clay, and with virtually no vegetation (Fig. 10B). The area, being in a space of 30 cm to 1 meter retreated under construction, was protected from direct sunlight, but it was indirectly lit, having no aphotic parts. The humidity was high and the animals were found in groups of 2 to 5 specimens.

**Figure 9.**
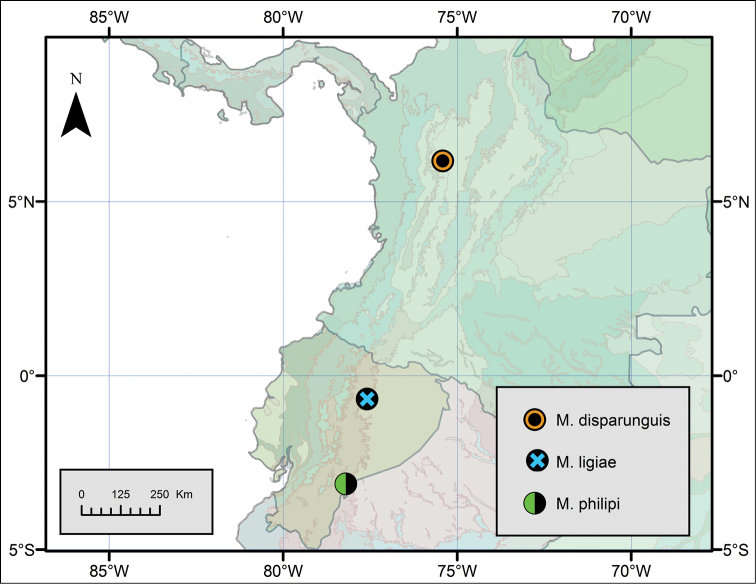
NW South America, showing the three *Metagovea* species occurring in the Andean Range. Shaded areas in the background are WWF ecoregions.

**Figure 10.**
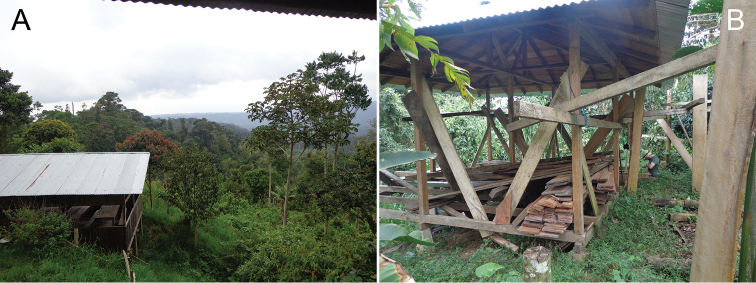
Ecuador, Pacto Sumaco, showing the collecting site. **A** General view. **B** Site where the type specimens were found. Photographs by A. B. Kury.

## Discussion

### Genital morphology of *Metagovea*

Comparing the score or so of published genital illustrations of Neotropical neogoveids, a few connecting traits can be advanced. Unfortunately male genitalia of *Metagovea disparunguis* are hitherto unknown.

Microtrichiae C may be either apical (short as in *Canga* and *Huitaca* or long as in *Metagovea* and *Tucanogovea*) or subdistal, shifted to dorsal as in *Brasiliogovea* and *Neogovea*. The apical pair of horns with associated shifting of microtrichiae C to dorso subdistal seem to be exclusive of *Neogovea* where they are long and well-developed and of *Brasiliogovea*, where they are much shorter and rounded. The apical margin of the spermatopositor in *Huitaca* is projected as a lobe with an augmented number of very short rod-like microtrichiae C placed in a tight row. *Canga* has only a convex apex, not nearly as projected as *Huitaca*, but with microtrichiae C equally reduced, although they do not form a row as in *Huitaca*.

Microtrichiae A are elongate and slender in most Neogoveidae, with the apparent exception of *Metagovea ligiae* and *Metagovea philipi*, where they are much shorter and thick. Curiously *Metagovea oviformis* does not match the pattern of *Metagovea*. The dma appear to be exclusive of *Metagovea ligiae* and *Metagovea philipi*, again absent in *Metagovea oviformis*. The paired dmm, which seem to be universal in neogoveids, appear to be fused to each other only in *Neogovea*.

### Diversity

*Metagovea*, now with four described species, displays a formal diversity far smaller than the real one, as shown by Benavides and Giribet (2007), who detected 17 undescribed species. This undersampling may be due to the small size of these animals, non-selective collecting and non-cyphophthalmid-focused collectors.

### Distribution

The distribution of the four species of *Metagovea* is disjunct. *Metagovea oviformis* occurs in the lowland forest in Amazon Basin (altitude 100 m), while the other three occur in the Central Andean Range (WWF NT0121 and NT 0136) in Ecuador and Colombia, at altitudes between 1150 and 2150 m. It is possible that *Metagovea oviformis* does not belong in *Metagovea*. This speculation is more tempting since a closely related genus has been described from Amazon basin. Benavides and Giribet (2007) already illustrated the distribution of a large number of undescribed species of Neogoveidae in NW South America. Here only the Andean species of *Metagovea* are represented in a Map (Fig. 9).

## Supplementary Material

XML Treatment for Metagovea

XML Treatment for Metagovea ligiae
